# Containment of a fatal and highly infectious disease outbreak

**DOI:** 10.11604/pamj.supp.2021.40.2.30897

**Published:** 2021-12-16

**Authors:** Wilbrod Mwanje, Muatsim Ahmed Mohammed, Erasmus Gidimadjor, Eva Mertens, Barbara Maria Bürkin, Axel Hoffmann

**Affiliations:** 1 Public Health Emergency Operations Center, Ministry of Health, Kampala, Uganda,; 2 African Field Epidemiology Network, Kampala, Uganda,; 3 National Public Health Laboratory (NPHL), National Tuberculosis Reference Laboratory (NTRL), Khartoum, Sudan,; 4 Municipal Health Directorate, Disease Control and Surveillance Unit, Ghana Health Service, Kpando, Ghana,; 5 Bernhard Nocht Institute for Tropical Medicine, Department of Infectious Disease Epidemiology, Bernhard-Nocht-Strasse 74, 20359 Hamburg, Germany,; 6 Global Partnership Initiated Biosecurity Academia for Controlling Health Threats (GIBACHT), Hamburg, Germany,; 7 Swiss Tropical and Public Health Institute, Department Education and Training, Basel, Switzerland,; 8 University of Basel, Basel, Switzerland,; 9 Global Partnership Initiated Biosecurity Academia for Controlling Health Threats (GIBACHT), Basel, Switzerland

**Keywords:** Case study, outbreak investigation, epidemiology, risk assessment, infection control

## Abstract

This case study was designed based on several experience with several viral haemorrhagic fevers (VHFs) outbreaks responded to in Uganda between 2000 and 2016. Fictitious scenarios have been included to facilitate learning of the users. The major goal of the case study is to facilitate learners to appreciate incident detection and the incident management processes for control and containment of a fatal and highly infectious viral disease outbreak. This case study is targeted towards health scientists of medicine, nursing, biomedical laboratory and public health background. We specifically orient learners on clinical presentation of viral infections and laboratory tests considered for incident detection, conducting a risk assessment for an infectious disease, Infection Prevention and Control in the outbreak setting, skills of incident management, analysis and interpretation of epidemiological data to aid epidemic response and control decisions.

## How to use this case study

### General instructions

This case study is best used as adjunct training materials to bolster concepts taught to learners during in-person training sessions. During the implementation of this case study 15 learners should be matched to one facilitator in a training room preferably at a round table. Each participant should be issued a copy of the case study prior to its implementation. The facilitator guide remains with the facilitator and should only be availed to the participants at the end of the case study implementation. The facilitator introduces the case study including learning objectives and makes mention of the requisite materials that participants should have at hand before starting the case study implementation. These materials will include the reference epidemic curve and calendar in annex 1 of the case study, a note book, pen and calculator. Participants should take turns to read the case scenario and the questions or tasks that immediately follow the scenario paragraphs. The reader on each turn should attempt to provide the answer or solution to the task. The task should then be opened up to the discussion of the rest of the group for concurrence on the most correct answers. The facilitator should make probes to stimulate participants to elucidate and arrive at the correct responses. Hints and facilitator notes are provided for use by the facilitator to steer the discussion in the direction which unlocks the puzzle. In the event that the solutions are not forthcoming the facilitator should provide them to the participants with reference to the facilitator´s guide.

### Audience

This case study has been designed to foster capacities among public health scientists including epidemiologists, doctors, nurses, biomedical laboratory scientists working at national departments of surveillance and public health emergencies, district surveillance departments and training institutions of field epidemiology programs.

### Prerequisites

By the time of implementing this case study, participants should have undergone training in Integrated disease surveillance and response, outbreak investigations, incident management processes and epidemiological data analysis and interpretation.

### Materials

Pen, notebook, calculator, epi-curve in annex 1 and calendar in annex 2.

### Level of training and associated public health activity

Intermediate public health surveillance officers in national, regional and or provincial public health surveillance departments and residents in Field Epidemiology Training Programs.

### Time required

Approximately 3.5 hours

### Language

English

## Case study material

- Download the case study student guide (PDF - 875 KB)
- Request the case study facilitator guide: contact info@gibacht.org
